# A contingent valuation analysis for assessing the market for genetically modified planting materials among banana producing households in Uganda

**DOI:** 10.1080/21645698.2020.1720498

**Published:** 2020-02-02

**Authors:** Enoch Mutebi Kikulwe, Marsy Asindu

**Affiliations:** a Development Impact Unit, Bioversity International, Kampala, Uganda; b Department of Agribusiness and Natural Resources Economics, Makerere University, Kampala, Uganda

**Keywords:** Banana, demand, genetic modification, GM commercialization, perceptions, Uganda

## Abstract

Banana is an important livelihood source for more than 12 million smallholder farmers in Uganda. Despite this contribution, its productivity continues to decline due to Banana Xanthomonas wilt (BXW). Cultural practices have been deployed to effectively control BXW but require a continuous and timely application, thus, prompting scientists to develop genetically modified (GM) bananas which display BXW resistance or tolerance. With prospects for commercialization of these GM bananas on the agenda, this paper applied a Contingent Valuation Method to assess producer acceptance of GM banana suckers among 233 banana producing households. Results show that producers were willing to pay between Ugandan shillings (UGX) 1100 to 1700 (US$0.28–0.44) per GM banana sucker Annual demand for GM banana suckers ranged from 70 to 82 million suckers. The results suggest that, in the event of not commercializing BXW-resistant GM bananas, Uganda loses an annual revenue ranging from UGX 76 to 139 billion (US$ 19.51 to 35.70 million).

## 1. Introduction

### 1.1. Banana Production, Banana Xanthomonas Wilt, and Biotechnological Solutions

Banana is an important staple crop in tropics with annual global production estimated to be about 145 million tons.^1^ Approximately a third of that production is in Africa, and Africa accounts for about 72% of the production of plantains.^1^ Investment in banana improvement technologies holds great potential for improving food security as these crops feed more people per unit area of production than other staple crops.^2^

In Uganda, Musa species (banana and plantain) are key crops supporting the livelihoods of millions of smallholder farmers,^3^ grown by than 75%. The Uganda’s agricultural census estimates 4 million tonnes of bananas to be grown on 807,000 ha, with approximately 68% of the crop produced in the Western Region, followed by the Central Region (23%), the Eastern Region (8%) and the Northern Region with less than 1%.^4^ Depending on the region, the daily per-capita consumption of bananas in Uganda is more than a 500 g cooking banana,^5^ conferring Uganda with the highest per-capita consumption of cooking banana in the world.^6^ Besides domestic consumption, the banana crop grown in Uganda contributes to farmers’ incomes through sales in fresh fruit and other value-added products such as chips, cakes, wines, juice, and flour.^7^ Moreover, different parts of the crop can also be used for other domestic and industrial purposes. Due to the increasing importance attached to bananas and the number of farmers benefiting from it, increasing productivity and profitability is seen as an important step toward achieving household and national food security and driving down the real price of food to accelerate economic growth, increase the sustainable management of natural resources, improve nutrition and health and reduce poverty and hunger.^8^

Despite its importance, years of devastating Banana Xanthomonas Wilt (BXW) infections have continued to cause massive losses in banana plantations thus reducing the food security of many households. The BXW disease, caused by *Xanthomonas vasicola pv. musacearum(Xvm)*, formely called Xanthomonas *campestris* pv. Musacearum^9^ starts with wilting of leaves or male bud and premature ripening of fruits, leading to the death of the whole plant. Where it occurs, BXW causes acute infections that can lead to the complete loss of a plantation. In the earlier years (2001 to 2004) of its onset, BXW caused about a 30–50% decrease in banana yields in Uganda.^10,11^ Recent studies, however, report yield losses resulting from BXW infections to be as high as 100% if not controlled.^8,12–14^ Meanwhile, economic losses of about US$2–8 billion have been reported over a decade as a result of the disease.^15^ As a result, average annual production declined from 10.5MT in 2002 to as low as 4.3MT in. 2016^16^

Considerable effort has been devoted to controlling BXW since its discovery in Uganda in 2001. Currently, two major approaches exist: first, the use of cultural control practices; and second, the use of genetic engineering (modification). The use of cultural practices includes avoiding introducing the disease into new areas by using clean planting materials, removing the male buds using a forked stick immediately after the banana bunch has formed its last cluster, removing all infected stems (by cutting off at soil level), and cleaning all used tools using sodium hypochlorite (JIK) or fire flame.^17^ However, BXW control using cultural practices can be inconsistent when the value-chain actors, especially the farmers and traders, fail to comply during implementation. Among these practices, the use of clean planting materials (such as tissue culture plantlets) is the least adopted practice because most farmers rely on planting materials (also known as suckers) from informal sources, and tissue-cultured plants are expensive. Besides, farmers do not have the technical means to verify whether the planting materials are BXW-free or not,^18^ yet long incubation periods of up to 24 months and latent infections have been reported.^19^ There is, therefore, a high risk of transmitting the disease within and across farms through infected planting materials, even when farmers apply the other control practices effectively. On the other hand, genetically modified (GM) BXW-resistant bananas have been developed through genetic engineering and field tested in Uganda.^20^ GM bananas aim to increase productivity and nutritional value, and could effectively contribute toward food security in the near future.^21^ Currently, Uganda’s experimental program with agricultural biotechnology is one of the largest in Africa,^5^ with reports showing some GM-lines could be released for multiplication, distribution, and commercialization in 2020.^8^

Evidence exists on the technical performance of the new technology against BXW as explained but nothing much has been documented as far as producer acceptance and demand for genetically modified banana planting material are concerned. Understanding producer acceptance for GM banana suckers and their perception toward the technology in this study will help inform policymakers and plant breeders on the market potential that exists, and the quantity of GM banana suckers needed to satisfy the potential market.

### 1.2. Producer Acceptance of GM Products

Genetically Modified (GM) food products in many countries have slowly been declared as GM food. However, this has come along with several issues pertaining to health, finance, and environmental safety,^22^ suggesting the need to understand producers’ perceptions toward GM crops.

GM organisms have been at the center of a major public controversy, involving different interests and actors. While much attention has been devoted to consumer views on GM food, there have been few attempts to understand the perceptions of GM technology among farmers in developing countries. In the context of these efforts and beyond, disproportionate attention has been given to the opinions of the end consumers of GM food compared to the views of the primary consumers of this technology.^23^ Farmers are directly affected by the spread of GM crops and have an important responsibility for their future. Their absence from the discussions and the limited academic interest in the views of farmers regarding GM crops amount to an important missing element in the debate and policymaking in the sector.^24^ Silencing of this group has also allowed other interest groups to speak on their behalf for their own ends. Organizations concerned with spreading the technology, for example, tend to emphasize the benefits of GM crops for farmers, often relying on absolute figures regarding the adoption of the technology in agriculture.^25^ For NGOs and social movements critical of GM crops, the promised benefits preached by advocates of GMOs are illusory, particularly for producers in developing countries.^26^

Despite this missing link, a number of scholars have recently stepped forward to document stories presenting producers’ perspectives in relation to GM crops. For instance, Mewius (2011) suggested that among some of the reasons for planting GM seeds are the increase in productivity, reduced pesticide use, obtaining a higher quality product and achieving better financial results,^27^ similarly, reported that the two greatest advantages associated with GM crops are their convenience of management and increased productivity. Fernandez et al.,^28^ go on to reveal that farmers in the US have continued to adopt GM cotton, soybeans and maize, citing their ability to increase yields as the primary reason and further asserts that this could lower the price of food, which would boost productivity in farming and increase the supply of food for the world’s rapidly growing population. In their study,^29^ also reported that farmers unanimously agreed that a reduction in the cost of herbicides was the main advantage of using GM crops, where nonselective herbicides are used. Celeres (2010)^30^, meanwhile, highlighted the ease of cultivation and crop management, weed control management, the increased strength and durability of storage as the key drivers of GM crop uptake by producers in USA.

According to declarations, the adoption of GM crops is also thought to reduce on-farm manual labor, especially with regard to the use of pesticides. However, Zambrano et al.,^31^ in their study revealed that widespread adoption of GM soy in the South, which requires less manpower during cultivation, led to the breakup of families and unemployment. In addition, producers seem increasingly convinced of the harmful effects of GMOs on health and the environment. They point to a series of socio-economic dangers that these plants represent for producers and consumers. They vigorously challenge the advantages stressed by advocates, arguing that the benefits claimed are illusory.^26^ As a result, farmers’ worries concerning GM crops go beyond financial and practical issues, despite these carrying more weight. The payment of royalties to the companies that own the technology and the ban they impose on replanting their seeds in ensuing seasons has on several occasions been mentioned as an important drawback in adopting GM varieties. Farmers also fear for the genetic crossover contamination of conventional crops, which may lead to financial penalties for the producer, and greater resistance of weeds to herbicides used in the growing process.^23,32^ Conversely, it is important to note that for some crop species, there is little or no risk of the recombined DNA leaking into the biome. Commercially grown bananas, for example, are predominantly sterile and do not produce pollen, so there is less chance of leakage. Pimbert et al.,^32^ also reveal that the spread of these crops will lead to a greater dependency of farmers on biotechnology. Of late, consumer resistance has also been identified as a major constraint to GM crop production by farmers.^33^ Clustered regularly interspaced short palindrome repeats (CRISPR) have been developed to counter such negative sentiments associated with a genetically modified organism. This is due to the fact that they are easier to design and implement, have a higher success rate, are more versatile and less expensive compared to the genetically modified organisms and other new breeding techniques(NBTs) such as zinc ﬁnger nucleases (ZFNs) and transcription activator-like effector nucleases (TALENs).^34^ Waltz et al.,^35^ also report in their study that such new breeding technologies (CRISPR), have brought about rapid positive changes in the attitude of many producers toward genetically engineered plants. However, the CRISPR technology is new and yet to be implemented in Uganda in the near future.

In terms of benefits, Ainembabazi et al.,^8^ conducted a study to understand future adoption and consumption of GM banana in the Great Lakes Region (GLA). Their findings revealed that each dollar invested in the development and dissemination of a GM resistant BXW banana in Uganda generates US$ 30 per plantlet, with an average adoption rate of 54%. The authors, however, anticipated that some undesirable attributes of the GM crop could affect the overall market potential. Considering all of the above, producer behavioral trends toward GM crops have become a vital factor influencing how lucrative and attractive the future market for GM crops will be. This will impact the future course of action for the public- and private-sector investments in the development and use of GM technology. Thus, producer and consumer acceptance toward GM technology is crucial for the global market of GM products, agricultural trade, and the future development of agricultural biotechnology.

## 2. Materials and Methods

### 2.1. Data

A face-to-face survey using a pre-tested questionnaire was conducted in August 2018 among banana-producing households in three administrative regions of Uganda (including Eastern, Central, and Southwestern), comprising three-distinct agro-ecological zones where cooking bananas (green bananas) are mostly grown and used as a source of staple food and income. The survey employed a simple random sampling procedure, in which each respondent had the same probability of being selected. Overall, 232 respondents were considered for this study. The first component of the survey involved collecting individual and household data. The second section contained perception and attitudinal questions regarding GM banana planting material. Lastly, the third section captured information on the respondent’s demand and willingness to pay for GM banana planting material.

For the ‘Willingness to Pay’ (WTP) section, enumerators first gave a brief description of GM banana planting material to the respondents before obtaining data on their WTP. This was done to create awareness since the product was new and not yet known by all respondents within the selected sites. After full description of the product to the respondent by the enumerator, through cheap talks, six bids were assigned randomly to different respondents (UGX 2500 (US$ 0.64), UGX3000 (US$ 0.77), UGX3500 (US$ 0.90), UGX4000 (US$ 1.03), UGX 4500 (US$ 1.16) and UGX 5000 (US$ 1.28)). The respondents were then asked if they were willing to pay for GM banana sucker at the randomly assigned bid price. Those who answered “yes” were asked if they would be willing to pay at another randomly assigned higher price that had been increased by 5%, 10%, 20%, 30%, 40%, or 50%. However, each respondent was only offered a single second bid. Respondents who answered “no” to the first bid were offered another randomly assigned lower price that was discounted by either 5%, 10%, 20%, 30%, 40%, or 50%.

### 2.2. Econometric Analysis

#### 2.2.1. Producer Acceptance of GM Planting Material

Producer acceptance of GM banana suckers was evaluated through their perception and attitudes. Producers’ perceptions were assessed for various GM-related parameters including its effect on the environment, health, food safety and risk and government and institutional regulation. Each perception response was measured on a five-point Likert scale with a score from (−1 for “strongly disagree,” −0.5 for “disagree,” 0 for “neutral,” 0.5 for “agree,” and 1 for “strongly agree”) as demonstrated by those of Kimenju & Hugo.^36^ However, for negative statements related to GM banana suckers, the scores were reversed with (−1 for strongly agree, −0.5 for agree, 0 for neutral, 0.5 for disagree and 1 for strongly disagree). Four categories of perception statements were created as indices for the study, including environment, health, food safety and risk, and government and institutional regulation. Each category constituted at least three perception statements, whose scores were later averaged to form an index (environment perception index, health perception index, food safety and risk perception index and government and institutional regulation perception index). Analysis of data then involved the use of descriptive statistics including means and standard deviations to characterize producers. T-tests were also carried out to establish any significant differences in perceptions between those who were aware of the GM banana suckers and those respondents who were not aware of it at all.

#### 2.2.2. Producer Demand and Willingness to Pay for GM Planting Material

A number of approaches are used in conducting the ex-ante assessment of new agricultural technologies. Some of the notable ones include Cost–Benefit Analysis (CBA), and several valuation methods, including Contingent Valuation, the Travel Cost Method, and Hedonic Pricing. For this study, double-bounded Contingent Valuation Method (CVM) was used since the GM banana planting material was still new and not yet in the markets (hypothetical product to farmers). Farmers were presented with two bids, with the second bid being contingent upon the response to the first bid. If the individual responded yes to the first bid, the second bid was greater; if it was a no to the first bid, then the second bid was smaller than the first one. Thus, there were four possible outcomes to the questions: (a) both answers are “yes”; (b) both answers are “no”; (c) a “yes” followed by a “no”; and (d) a “no” followed by a “yes”.^37^ The four probabilities are then denoted as follows:

(1) $$Pryy\left(B,B^{u}\right)=\mathrm{Pr}\left[B\leq WTP,B^{u}\leq WTP\right]=\mathrm{Pr}\left[B\leq WTP|B^{u}\leq WTP\right]\mathrm{Pr}\left[B^{u}\leq WTP\right]=\mathrm{Pr}\left[B^{u}\leq WTP\right]=1-F\left(B^{u}\right)$$

(2) $$Pryn\left(B,B^{u}\right)=\mathrm{Pr}\left[B\leq WTP<B^{u}\right]=F\left(B^{u}\right)-F\left(B\right)$$

(3) $$Prny\left(B,B^{d}\right)=\mathrm{Pr}\left[B^{d}\leq WTP<B\right]=F\left(B\right)-F\left(B^{d}\right)$$

(4) $$Prnn\left(B,B^{d}\right)=\mathrm{Pr}\left[B>WTP,B^{d}>WTP\right]=F\left(B^{d}\right)$$

where Pryy is the probability of answering “yes” “yes,” Pryn is the probability of answering “yes” “no,” Prny is the probability of answering “no” “yes,” and Prnn is the probability of answering “no” “no;” B is the price in the first question, $B^{u}$ is the higher price in the second question; WTP is the Willingness to Pay, and F is the Cumulative Distribution function (CDF). Combining the probabilities of the four outcomes, the log-likelihood function (*lnL*) for the sample then takes the form:

(5) $$\begin{array}{rl} & lnL=\sum_{1}^{N}\left\{yy_{i}lnPr_{yy}\left(B_{i,}B_{i}^{U}\right)\right.\\ & \;\;\;\;\;\;\;\;\;\;\;\;\;\;\;\;+yn_{i}lnPr_{yn}\left(B_{i,}B_{i}^{U}\right)\\ & \;\;\;\;\;\;\;\;\;\;\;\;\;\;\;\;+ny_{i}lnPr_{ny}\left(B_{i,}B_{i}^{d}\right)\\ & \left.\;\;\;\;\;\;\;\;\;\;\;\;\;\;\;\;+nn_{i}lnPr_{nn}\left(B_{i,}B_{i}^{d}\right)\right\} \end{array}$$

where yy, yn, ny and nn are dummy variables, that is, yy=1 if respondent says yes – yes (yy) for the two questions, otherwise will be zero. Thus, the mean WTP was then evaluated using the following equation as adopted from Shultz & Soliz:^38^

(6) $$WTP=\beta_{0}+\left(\sum^{\beta_{2}}\times_{2}\ldots \ldots \ldots \ldots \ldots \beta_{k}\times_{k}\right)/-\beta_{1}$$

where: $\beta_{0}$ is the estimated constant, $\beta_{k}$ are the estimated co-efficient parameters, $\times_{k}$ are the mean values of the explanatory variables and $\beta_{1}$ is the estimated co-efficient of the Bid. A bivariate probit model was later fitted to assess the factors that influence producers’ willingness to pay (WTP) for GM banana suckers.

#### 2.2.3. Estimation of Market Potential for GM Banana Plating Material

The estimation of market potential for a product is critical in evaluating its viability as it provides an estimate of the maximum total sales potential for a given market.^39,40^ Once the estimated market potential has been calculated, it would be possible to determine if the market is large enough to sustain the proposed production or sustain an additional producer in the market place.^40^ Market potential (demand) for GM banana suckers was estimated in terms of maximum annual total sales revenue that could be generated from GM banana suckers from the three regions under study. The following formula derived by^40^ was used to estimate the market potential.

(7) MP=N×P×A

Where: MP = is the market potential, N = Number of possible buyers at price P, *P* = Mean Willingness to pay or average selling price and A = Average annual consumption.

## 3. Results and Discussion

### 3.1. Socioeconomic Characteristics

Individual and household characteristics of respondents are shown in Table 1. For purpose of conducting a robust description of the banana producers, the analysis of socioeconomic characteristics involved the formation of two categories of banana-producing households (1) self-insufficient producers whose own consumption is supplemented by some banana purchased from the market, and (2) self-sufficient producers who only consume what they produce. The self-sufficient banana producers were slightly more educated than the self-insufficient producers (see Table 1). As expected, only the self-insufficient producers spent money on the purchase of bananas for household consumption. More of the self-sufficient producers were involved in banana sales as opposed to their counterparts. This difference in their involvement in banana sales is attributed to the fact that most of them have surplus production to meet consumption and marketing needs. The two producer categories perceived that lack of improved pest and soil fertility management technologies is a huge hindrance to banana production with the self-insufficient producers being more affirmative as reflected by the Soil fertility and pest management technology index presented on Table 1.

**Table 1.** Socioeconomic characteristic of respondents.

Respondent characteristics	Self-insufficient producers n = 145	Self-sufficient producers n = 87	Total	t-value
Age of the respondent (years)	46.95 (14.46)	47.94 (15.92)	47.32 (15.00)	0.49
Annual expenditure on bananas (UGX)	452865.50 (464055.30)	0.00 (0.00)	283040.90 (427221.10)	−9.09***
Household size (Number of persons)	5.49 (2.46)	5.39 (2.48)	5.45 (2.46)	−0.30
Health characteristic index	0.05 (0.06)	0.06 (0.71)	0.06 (0.70)	0.12
Male respondents (%)	46.21	55.17	49.57	1.32
Respondent’s education level (years)	7.32 (4.40)	8.34 (3.91)		4.44*
Married respondents (%)	71.03	73.56	71.98	0.41
Harmful environmental effect index	0.36 (0.68)	0.41 (0.52)	0.38 (0.62)	0.65
Involvement in banana sales (%)	25.00	67.47	40.53	6.87***
Awareness of GM products (%)	26.21	26.44	26.29	0.04
Religious influence in decision making (%)	5.52	5.75	5.60	0.07
Area under banana production (Acres)	1.04 (1.72)	2.94 (6.14)	1.75 (4.09)	3.50***
Access to credit (%)	6.21	9.20	7.33	0.84
Access to extension services (%)	4.83	8.05	6.03	0.99
Experience in banana production (Years)	15.77 (13.89)	20.02 (14.87)	17.36 (14.38)	4.97**
Distance to banana market (Km)	2.76 (3.94)	3.13 (4.84)	2.90 (4.29)	0.64
Soil fertility and pest management technology index	0.63 (0.04)	0.44 (0.06)	0.56 (0.03)	−2.77***

Note: Numbers in parentheses are standard deviations. *, **, *** denote significance at 10%, 5% and 1% levels, respectively.

Self-sufficient producers were also characterized by large acreages of land, equivalent to an average area threefold that of self-insufficient producers allocated to banana production. Self-sufficient producers have more years of banana farming experience than the self-insufficient producers. Overall, producers had an average age of 47 years, with an average household size of approximately 5 people. No significant differences were observed based on the gender and age of the producer.

### 3.2. Producer Perceptions and Acceptance of GM Banana Planting Material

Banana producers’ perceptions were considered very important to the process of making GM banana planting material acceptance decisions. Perceptions were generally compared across two producer groups: (1) those who were aware of GM technology, that is, those who have received any kind of information or have already heard about anything related to GM technologies, comprising of 27%, and (2) those who were not aware of GM technologies, making up 73% of the sample. The low awareness of GM technology among producers is in line with Tanius & Seng,^41^ who found out that the awareness level of consumers toward GM food concept was still low. Similarly, Chen and Chern,^41^ also showed in their study that the majority of consumers are still not very well-informed about the GM foods.

Table 2 shows that mostly negative producers’ perceptions on the interaction between GM and the environment, with an exception regarding the belief that GM technology will eradicate crop pests and diseases among producers who were not aware or had not heard anything related to GM technology. Producers who were aware of GM technologies were opposed to the idea of eating GM-derived material, as this was deemed harmful to them and their families. Likewise, they perceived that if anything went wrong with GM technology, a global disaster would occur. On the contrary, both types of producers held their positive view that if most of the population is in support of GM, then it should be legalized. Banana producers also believed that the government is doing sufficient to regulate GM use in Uganda.

**Table 2.** Producer perceptions and acceptance of GM banana planting materials.

Perception statement	Aware of GM technology	Not aware of GM technology	Overall mean score	t-value
Environment				
Humans interference with nature will result into disaster	−0.78 (0.37)	−0.65(0.57)	−0.69 (0.53)	1.58
Through GMO, humans are harshly abusing the environment	−0.84 (0.36)	−0.63 (0.57)	−0.68 (0.53)	2.79***
Pesticides and fertilizer use are harmful to the environment	−0.50 (0.60)	−0.48 (0.66)	−0.48 (0.65)	0.21
GM technology will eradicate Crop pests and diseases	−0.03 (0.71)	0.19 (0.64)	0.13 (0.67)	2.69**
Harmful environmental effects of GM will surface in future	−0.43 (0.59)	−0.36 (0.64)	−0.38 (0.62)	0.68
Even if GM food is advantageous, it is basically against nature	−0.53 (0.53)	−0.27 (0.70)	−0.34 (0.67)	2.69***
Environment perception index	−0.52 (0.34)	−0.37 (0.37)	−0.41 (0.37)	2.80***
Health				
GM additives are not harmful to health	0.00 (0.69)	0.10 (0.73)	0.08 (0.72)	0.96
Harmful health effects of GM are likely to appear in future	−0.47 (0.61)	0.68 (0.68)	−0.35 (0.66)	1.60
Eating GM food will harm me and my family	−0.12 (0.71)	0.08 (0.70)	0.03 (0.70)	1.99**
GM technology should not be used even for medical purposes	−0.03 (0.70)	0.09 (0.69)	0.06 (0.69)	1.20
Health perception index	−0.16 (0.42)	−0.01 (0.43)	−0.05 (0.43)	2.33**
Food safety and risk				
Food safety and nutrition labels can be trusted	0.12 (0.66)	0.13 (0.68)	0.13 (0.67)	0.09
Risks associated with food safety can be avoided	0.20 (0.64)	0.32 (0.62)	0.29 (0.63)	1.31
Risks impacting food safety are very important	−0.66 (0.38)	−0.66 (0.48)	−0.66 (0.46)	0.09
Anything gone wrong with GM, will result into global disaster	−0.68 (0.44)	−0.54 (0.58)	−0.57 (0.55)	1.77*
Food safety and risk perception index	−0.26 (0.30)	−0.19 (0.33)	−0.20 (0.33)	1.46
Government and Institutional regulations				
If majority of people favor GM, it should be legalized	0.47 (0.64)	0.64 (0.50)	0.59 (0.54)	2.12**
Government effectively monitors GM use	0.02 (0.71)	0.15 (0.71)	0.11 (0.71)	1.23
Government should spend more to increase food safety	−0.81 (0.33)	−0.81 (0.36)	−0.81 (0.35)	0.03
Government and institutional regulation perception index	−0.11 (0.35)	−0.01 (0.35)	−0.04 (0.35)	1.91*

Note: Numbers in parentheses are standard deviations. *, **, *** denote significance at 10%, 5% and 1% levels, respectively.

Generally, the findings still reflect negative producers’ perception of the GM technology in relation to the purported threat of adverse influence on health, environment, and food safety, similar to Kikulwe et al. (2011)^43^, with those aware of the technology being even more negative compared to those who are unaware. This finding could be attributed to negative information being disseminated in public by anti-GM advocates. This implies that there is a great need for stakeholders involved in GM technology deployment and promotion to provide, sensitize, and disseminate adequate and relevant science-based information concerning GM to the general public.

### 3.3. Willingness to Pay for GM Banana Planting Material

A bivariate probit model was estimated to assess the mean willingness to pay value for GM banana suckers. The results showed that the mean willingness to pay per GM banana sucker ranged from Ugandan shillings (UGX) 1092 to UGX1702 (US$ 0.28 to 0.44) per sucker. The Western region had the highest percentage (80%) of respondents willing to pay for suckers at the mean price of UGX 1092 (US$ 0.28) per GM banana planting material, while the Eastern region had the least (44%) willing to pay for the GM banana planting material. At the upper mean willingness to pay price (UGX 1702- US$ 0.44), the percentage of respondents willing to pay for GM banana sucker dropped in all the three regions (Table 3).

**Table 3.** Percentage of respondents willing to pay for GM planting materials by region.

Region	Percentage of Households WTP at UGX 1092 mean price and above per GM banana sucker	Percentage of Households WTP at UGX 1702 and above per GM banana sucker
Central	45.5	38.5
Western	80.0	77.5
Eastern	43.8	38.8

### 3.4. Factors Influencing Producers’ WTP for Genetically Modified Banana Planting Material

Data for the banana producers were estimated in a bivariate probit model to obtain the factors that influenced producer willingness to pay for GM banana planting materials. The results of the modeling efforts are as shown in Table 4.

**Table 4.** Bivariate probit results for factors influencing producers’ WTP for GM banana planting materials.

Variable	Marginal effects	Standard Error
Bid price 1	0.0000*	0.0000
Bid price 2	0.0000**	0.0000
Age of the respondent (years)	0.0006	0.0016
Health safety characteristic index	0.0276	0.0369
Harmful environmental effect index	−0.0588	0.0409
Annual expenditure on bananas (UGX)	0.1493**	0.0601
Involvement in banana sales (1 = Yes, 0 = No)	0.0422	0.0536
Household size (Persons)	−0.0143	0.0097
Soil fertility and pest management technology index	−0.1878***	0.0507
Awareness on GM products (1 = Yes, 0 = No)	−0.0245	0.0554
Religious influence in decision making (1 = Yes, 0 = No)	−0.1643	0.1224
Area under banana production (Acres)	0.0245**	0.0120
statistics		
Number of observations		216.0000
Wald chi2(22)		41.6200
Prob > chi2		0.0070
Log likelihood		−238.7417

Note: Numbers in parentheses are standard deviations. *, **, *** denote significance at 10%, 5%, and 1% levels, respectively.

Bid prices as usual had a negative and statistically significant influence on producers’ willingness to pay for GM banana suckers. This implies that an increase in the amount of the bid price offered by UGX 1 reduced the likelihood of a producers’ willingness to pay for GM banana suckers at less than 10%. This finding perfectly mimics the theory of demand, which shows an inverse relationship between price and quantity demanded of a commodity.

The soil fertility and pest management technology perception index stood out to be the most important factor influencing producers’ willingness to pay decisions for GM suckers. An increase in soil fertility and pest management technology perception index by one unit decreased the likelihood of a producer’s willingness to pay for GM suckers by 0.1878. This could because farmers perceive their indigenous varieties to be more tolerant to soil deficiency and pest and disease infections than improved or new varieties. This finding is in line with Kansiime and Mastenbroek,^44^ who reported that farmers considered their local crop varieties to be more adaptable to drought and pests compared to improved and modified varieties.

On the other hand, annual expenditure on bananas and the total area of land allocated to banana production had a positive and significant influence on producers’ willingness to pay for GM banana suckers. An increase on annual expenditure on bananas by UGX 1 increased the likelihood of the producers’ willingness to pay for GM banana suckers by 0.1493. It was noted that a positive correlation exists between annual expenditure on bananas and annual income. Individuals with high annual income earnings were likely to be willing to purchase GM banana suckers. This finding resonates with those of Kimenju & De–Groote,^36^ who assert that income influences willingness to pay positively. A unit increase in the area under banana production as well increased the probability of the producer willingness to pay for GM banana suckers by 0.0245. This is consistent with the findings of Schnurr & Addison,^5^ that show that the larger the farm, the more likely respondents are to hold positive attitudes toward GM crops.

### 3.5. Market Potential for GM Banana Planting Materials

Demand and market potential for GM banana suckers were estimated based on information extracted from the Uganda census of Agriculture report that was documented by Uganda Bureau of Statistics.^4^ The number of households engaged in banana farming was estimated at 459,555 in central, 696,102 in western and 209,283 in eastern Uganda, respectively. Out of these households, on average, 56% of the sample was willing to pay the price of UGX 1092 (US$ 0.28) and above per GM banana sucker. At the regional level, 45% of the households in the Central, 80% in the Western and 44% in the Eastern were willing to pay the mean price of UGX 1092 (US$ 0.28) and above per GM banana sucker. This results into a potential buying population of 209,098, 556,882 and 91,561 for the Central, Western, and Eastern regions, respectively. On the other hand, at the mean willingness to pay the value of UGX 1702 (US$ 0.44) and above per GM banana sucker, 52% of the household on average were willing to pay. Specifically, 39% of the households in Central, 78% in the Western and 39% in the Eastern regions were willing to pay that mean price and above. This resulted in an equivalent buying population of 176,929, 539,479 and 81,094 for the Central, Western, and Eastern regions, respectively. The average proportions of 56% and 52% households willing to pay for GM banana sucker at the mean price of 1092 (US$ 0.28) and 1702 (US$ 0.44) and above, respectively, is in line with the mean adoption rate of 55% reported by Ainembabazi et al.^8^

Using the generated statistics, the overall potential annual demand for each region was then derived by multiplying the average potential number of GM banana suckers demanded annually per household for each region by the number of households willing to pay at mean prices and above. The market potentials were computed by multiplying the overall potential annual quantities of GM banana suckers demanded for each region by the mean willingness to pay prices.

From the computations, the overall potential annual quantity demanded of GM banana suckers stood at around 70 and 82 million suckers at the mean price of UGX 1092 and 1702, respectively. The Western region had the highest potential annual demand for GM banana planting material being a major production hub in the country, while the Eastern region had the least overall potential annual demand for GM banana suckers.

The total market potential for GM banana suckers was UGX 76 billion (US$ 19.51 million) at the mean price of UGX 1092 (US$ 0.28) and UGX 139 billion (US$ 35.70 million) at a mean price of UGX 1702 (US$ 0.44) (see Table 5). This implies that for an entrepreneur intending to skim the market, a price of UGX 1702 (US$ 0.44) per GM sucker would be the most convenient. But if the motive of the entrepreneur is to penetrate the market; then, the mean price of UGX 1092 (US$ 0.28) per GM banana planting material would be appropriate though he or she would have to forgo an extra UGX63 billion (US$ 16.18 million) that would be realized if the skimming option was considered.

**Table 5.** Producer demand and market potential for GM banana planting materials.

Variable	Central	Western	Eastern	Overall
Number of potential buying households				
GM banana planting material 1	209098	556882	91561	857540
GM banana planting material 2	176929	539479	81097	797505
Mean willingness to pay price (UGX)				
GM banana planting material 1	1092	1092	1092	1092
GM banana planting material 2	1702	1702	1702	1702
Mean annual demand per Household				
GM banana planting material 1	129	65	73	
GM banana planting material 2	189	77	83	
Total annual demand (Millions)				
GM banana planting material 1	27	36	7	70
GM banana planting material 2	33	42	7	82
Market potential (Billion UGX)				
GM banana planting material 1	29.5	39.5	7.3	76.3
GM banana planting material 2	56.9	70.7	11.5	139.1

Note: GM banana planting materials 1&2 means the planting materials at mean prices of UGX 1092 and UGX 1702 respectively. (Exchange rate: 1 US$ is equivalent to UGX 3894)

## 4. Conclusion

This study assessed acceptance of GM planting material among banana-farming households in Uganda. The findings show that more than 62% of banana producers are self-insufficient with about one acre of land allocated to banana production. The producers genuinely agree that if the majority of the population is in support of GM, it should be legalized. Apparently, a huge market potential, valued at a tune of UGX 76 billion (US$ 19.51 million) to 139 billion (US$ 35.70 million) for GM banana suckers have been estimated. However, some negative perceptions that may affect the overall market potential and future acceptance of GM products were also elicited from the producers. Such concerns were mainly related to possible threats that GM products may pose to health, environment, food safety and risk and costs linked to government and institutional regulations.

The existence of these negative perceptions, therefore, points to the need to conduct sensitization campaigns for banana producers and masses at large on GM technology as most of the information that ignites the negative perceptions are mainly false and not based on scientifically proven evidence. Similarly, new breeding technologies (NBTs) such as CRISPR have shown positive results, and recent studies, for example, Waltz,^35^ have revealed a rapid positive change in attitude toward genetically engineered plants using CRISPR-editing. Thus, a study looking at how farmers in Uganda perceive CRISPR and NBTs and how this affects their attitudes and perceptions toward genetic engineering technology needs to be undertaken in the future. Finally, to effectively predict the market, there is also a need to carefully project the market potential for GM banana planting materials beyond 1 year. However, since the crop is vegetatively propagated, on average each planting material procured in year one will produce at least six extra suckers for use in the subsequent year. This implies that the projected first-year market has a possibility of either declining or expanding in subsequent years.

## References

[1] FAOSTAT. Agriculture data. 2014 Accessed 2019 130. http://faostat.

[2] West PC, Gerber SJ, Engstr PM. Leverage points for improving global food security and the environment. Science. 2014;345:325–28. doi:10.1126/science.1246067. 25035492

[3] Nowankunda K, Ngambeki D, Tushemereirwe WK. Increasing small-scale farmers’ competitive in banana (Musa spp.) production and marketing. Acta Hortic. 2010;879:759–66. doi:10.17660/ActaHortic.2010.879.82.

[4] UBOS. Summary report on uganda census of agriculture, 2008/2009. 2010. www.ubos.org.

[5] Schnurr MA, Addison L. Which variables influence farmer adoption of genetically modified orphan crops? Measuring attitudes and intentions to adopt GM matooke banana in Uganda. AgBioForum. 2017;20:133–47.

[6] Clarke T Banana lab opens in Uganda: genetic modification of clonal crop could soon follow. 2003.

[7] Karamura DA. Numerical taxonomic studies of the East African highland bananas (Musa AAA-East Africa) in Uganda. A thesis submitted for the degree of Doctor of Philosophy, Department of Agricultural Botany, University of Reading. 1998. p. 189.

[8] Ainembabazi JH, Tripathi L, Rusike J, Abdoulaye T, Manyong V. Ex-ante economic impact assessment of genetically modified banana resistant to Xanthomonas wilt in the great lakes region of Africa. PLoS One. 2015;10(9):e0138998. doi:10.1371/journal.pone.0138998. 26414379 PMC4587572

[9] Nakato VG, Christelova P, Were E, Nyine M, Coutinho AT, Dolezel J, Uwimana B, Swennen R, Mahuku G. Sources of resistance in musa to Xanthomonas campestris PV musacearum, the causal agent of banana xanthomonas wilt. Plant Pathol. 2019;68:49–59. doi:10.1111/ppa.12945.

[10] Karamura E, Kayobyo G, Blomme G, Benin S, Eden-Green JS, Markham R Impacts of BXW epidemic on the livelihoods of rural communities in Uganda. P. 57 in Proceedings of the 4th international bacterial wilt symposium; 2006 7 17–20; York, UK Central Science Laboratory. 2006.

[11] Kalyebara MR, Ragama PE, Kagezi GH, Kubiriba J, Bagamba F, Nankinga KC, Tushemereirwe wk. Economic Importance Of The Banana Bacterial Wilt in Uganda. Afr Crop Sci J. 2006;14:93–103

[12] Blomme G, Dita M, Jacobsen KS, Pérez Vicente L, Molina A, Ocimati W, Poussier S, Prior P. Bacterial diseases of bananas and enset: current state of knowledge and integrated approaches toward sustainable management. Front Plant Sci. 2017;8:1290. doi:10.3389/fpls.2017.01290. 28785275 PMC5517453

[13] Blomme G, Jacobsen K, Ocimati W, Beed F, Ntamwira J, Sivirihauma C, Ssekiwoko F, Nakato V, Kubiriba J, Tripathi L, et al. Fine-tuning banana Xanthomonas wilt control options over the past decade in east and central Africa. Eur J Plant Pathol. 2014;139:271–87. doi:10.1007/s10658-014-0402-0.

[14] Ocimati W, Bouwmeester H, Groot JCJ, Tittonell P, Brown D, Blomme G. The risk posed by Xanthomonas wilt disease of banana: mapping of disease hotspots, fronts and vulnerable landscapes. PLoS One. 2019;14(4):e0213691. doi:10.1371/journal.pone.021. 30939129 PMC6445462

[15] Tripathi L, Mwangi M, Abele S, Aritua V, Tushemereirwe WK, Bandyopadhyay RA. A threat to banana production in east and central Africa. Plant Dis. 2009;93:440–51. doi:10.1094/PDIS-93-5-0440. 30764143

[16] FAOSTAT. 2016 data on bananas and plantains from FAOSTAT - production/crops. 2016

[17] Kubiriba J, Karamura EB, Jogo W, Tushemereirwe WK, Tinzaara W. Community mobilization: A key to effective control of banana xanthomonas wilt. J Dev Agric Econ. 2012;4(5):125–31. doi:10.5897/JDAE11.098.

[18] Jogo W, Karamura E, Tinzaara W, Kubiriba J, Rietveld A. Determinants of farm-level adoption of cultural practices for banana xanthomonas wilt control in Uganda. J Agric Sci. 2013;5:70–82. doi:10.5539/jas.v5n7p70.

[19] Ocimati W, Ssekiwoko F, Karamura E, Tinzaara W, Eden-Green S, Blomme G. Systematicity of Xanthomonascampestris PV. musacearum and time to disease expression after inflorescence infection in East African highland and pisang awak bananas in Uganda. Plant Pathol. 2013;62(4):777–85. doi:10.1111/ppa.2013.62.issue-4.

[20] Namukwaya B, Tripathi L, Tripathi NJ, Arinaitwe J, Mukasa SB, Tushemereirwe WK. Transgenic banana expressing Pflp gene confers enhanced resistance to Xanthomonas wilt disease. Transgenic Res. 2012;21:855–65. doi:10.1007/s11248-011-9574-y. 22101927

[21] Siddhesh G, Thumballi G. Genetically modified bananas: to mitigate food security concerns. Sci Hortic. 2017;214:91–98. doi:10.1016/j.scienta.2016.11.023.

[22] Bashir I, Golnaz R, Zainal AM, Juwaidah S Malaysian consumers’ awareness and GM food: what are the factors influencing? 4th International Conference on Business and Economic Research Proceeding; 2013; Bandung, Indoneisa. (pp. 549–61). doi:10.1177/1753193413492058.

[23] Hall C. Identifying farmer attitudes towards genetically modified (GM) crops in Scotland: are they pro- or anti-GM? Geoforum. 2008;39:1204–12. doi:10.1016/j.geoforum.2007.06.003.

[24] Guehlstorf NP. Understanding the scope of farmer perceptions of risk: considering farmer opinions on the use of Genetically Modified (GM) crops as a stakeholder voice in policy. J Agric Environ Ethics. 2008;21:6541–58. doi:10.1007/s10806-008-9116-7.

[25] Monsanto. O que fazemos [what we do]. 2014. http://www.monsanto.com/global/br/produtos/pages/default.aspx

[26] Almeida C, Massarani L. Farmers prevailing perception profiles regarding GM crops: a classification proposal. Public Underst Sci. 2018;27(8):952–66. doi:10.1177/0963662518766281. 29616889

[27] Kruger M, Van Rensburg JBJ. Van den Berg J. Transgenic Bt maize farmer's perceptions, refugee compliance and reports of stem borer resistance in south africa. J Appl Entomol. 2012;136:38–50.

[28] Fernandez-Cornejo J, Wechsler S, Livingston M, Mitchell L EconomicResearch Report Number 162: genetically engineered crops in the United States. Economic Research Service, United States Department of Agriculture; 2014 2. Available from: http://www.ers.usda.gov/media/1282246/err162.pdf.

[29] Massarani L, Polino C, Cortassa C, Fazio ME. Vara AM. O que pensam os pequenos agricultores da Argentina sobre os cultivos geneticamente modificados? Ambiente & Sociedade. 2013;16(3):1–22.

[30] CELERES. Os beneficios socioambientais da biotechnologia Agricola no Brasil: 1996 – 2009. Uberlandia Celeres Ambiental. 2010. Disponivel em: http://www.celeresambiental.com.br/pdf/Rel_BiotechBenefits_2009_Ambiental.pdf

[31] Zambrano P, Maldonado JH, Mendoza SL Women cotton farmers their perceptions and experiences with transgenic varieties. International Food Policy Research Institute; 2011

[32] Pimbert M, Wakeford T, Satheesh PV. Pequenos agricultores e marginalizados rurais expressamsesobre a agricultura e os OGM. In: Zanoni M, Ferment G, editors. Transgênicos para quem? Agricultura Ciência Sociedade [Transgenic for whom? Agriculture Science Society]. Brasília (Brazil): Ministério do Desenvolvimento Agrário; 2011. p. 406–19.

[33] Panzarini HN, Bittencourt MVJ, De Aville Mantos SAE, Wosaick AP. Biotechnology in agriculture: the perceptions of farmers on the inclusion of Genetically Modified Organisms (GMOs) in agricultural production. Afr J Agric Res. 2015;10(7):631–36. doi:10.5897/AJAR2014.9323.

[34] Borrelli VMG, Brambilla V, Rogowsky P, Marocco A, Lanubile A. The enhancement of plant disease resistance using CRISPR/Cas9 technology. Front Plant Sci. 2018;9:1245. doi:10.3389/fpls.2018.01245. 30197654 PMC6117396

[35] Waltz E. With a free pass, CRISPR-edited plants reach market in record time. Nat Biotechnol. 2018;36(1):6–7. doi:10.1038/nbt0118-6b. 29319694

[36] Kimenju SC, Hugo DG Consumers’ Willingness to pay for genetically modified food in Kenya. Paper presented at: the 11th International Congress of the EAAE (European Association of Agricultural Economists; 2005; Copenhagen, Denmark. The Future of Rural Europe in the Global Agri - Food system.

[37] Hanemann MW, Loomis JB, Kannimen BJ. Statistical efficiency of double-bounded dichotomous choice contingent valuation. Am J Agric Econ. 1991;73(4):1255–63. doi:10.2307/1242453.

[38] Shultz S, Soliz B. Stakeholder willingness to pay for watershed restoration in rural bolivia. J Am Water Resour Assoc. 2007;43(4):947–56. doi:10.1111/j.1752-1688.2007.00076.x.

[39] Lehmann DR, Winer RS. Analysis for marketing planning. 6th ed. New York, NY: McGraw-Hill Companies, Inc. 2005. p. 170–208.

[40] Wolfe K Estimating market potential check-list. center for agribusiness and economic development report. The University of Georgia; 06–089, 2006. Available from http://www.caed.uga.educ/publications/2006/pdf/CR-06-08.pdf. 2006.

[41] Tanius E, Seng WS. Consumers awareness towards genetically modified (gm) foods. Int J Bus Econ Law. 2015;6:17–26.

[42] Chen H, Chern WS Consumer acceptance of genetically modified foods. Paper presented at: the The Annual Meeting of the American Agricultural Economics Association (AAEA);2002 7 28–31; Long Beach, California

[43] Kikulwe ME, Wesseler J. Falck-zepeda J. Attitudes, Perceptions and Trust. Insights from a Consumer Survey regarding Genetically Modified Banana in Uganda. Appetite. 2011;57:401–413. 21704665 10.1016/j.appet.2011.06.001

[44] Kansiime KM, Mastenbroek A. Enhancing resilience of farmer seed system to climate-induced stresses: insights from a case study in West Nile region, Uganda. J Rural Stud. 2016;47(2016):220–30. doi:10.1016/j.jrurstud.2016.08.004.

